# A Novel Adsorbent Magnetic Graphene Oxide Modified with Chitosan for the Simultaneous Reduction of Mycotoxins

**DOI:** 10.3390/toxins10090361

**Published:** 2018-09-06

**Authors:** Atena Abbasi Pirouz, Roghayeh Abedi Karjiban, Fatimah Abu Bakar, Jinap Selamat

**Affiliations:** 1 Department of Food Science, Faculty of Food Science and Technology, Universiti Putra Malaysia, Serdang, 43400 Selangor, Malaysia; atenapirouz.upm@gmail.com (A.A.P.); Fatim@upm.edu.my (F.A.B.); 2 Institute of Tropical Agriculture and Food Security, Universiti Putra Malaysia, Serdang, 43400 Selangor, Malaysia; 3 Department of Chemistry, Faculty of Science, Universiti Putra Malaysia, Serdang, 43400 Selangor, Malaysia; roghayeh@upm.edu.my

**Keywords:** adsorbent, reduction of mycotoxins, isotherms, kinetics, thermodynamics

## Abstract

A novel magnetic graphene oxide modified with chitosan (MGO-CTS) was synthesised as an adsorbent aimed to examine the simultaneous removal of mycotoxins. The composite was characterised by various procedures, namely Fourier-transform infrared spectroscopy (FTIR), X-ray diffraction (XRD) and a scanning electron microscope (SEM). The adsorption evaluation was considered via pH effects, initial mycotoxin concentration, adsorption time and temperature. Adsorption isotherm data and kinetics experiments were acquired at the optimum pH 5 fit Freundlich isotherm as well as pseudo-second-order kinetic models. The thermodynamic results indicated that the adsorption of the mycotoxins was spontaneous, endothermic and favourable.

## 1. Introduction

Mycotoxins are produced as the secondary metabolites of fungal species and can exert toxic effects on animals and humans [1]. The global contamination of foods and feeds by mycotoxins has become a great challenge for researchers. Up to now, more than 400 different mycotoxins have been identified; the foremost categories of mycotoxins influencing feedstuffs are aflatoxinB_1_ (AFB_1_), ochratoxinA (OTA) and zearalenone (ZEA) [2]. This was reported from surveillance for detecting mycotoxins in cereals and animal feeds, whereby more than one toxin can occur in the same commodity. Occurrences of mycotoxins have synergistic or additive effects [3]. The most common combination of AFB_1_, OTA and ZEA is associated with a variety of toxic effects, chronic and acute in animals and humans. Therefore, their presence is one of the most relevant and worrisome problems among feedstuffs [4]. Mycotoxin contamination has created a major concern, and the reduction of mycotoxins from feedstuffs is necessary. Many attempts have been made to detoxify mycotoxins, including biological methods, chemical treatments, and adsorption techniques. Among the methods used for treatment, adsorption is the most effective [5]. Several studies have shown the applicability of adsorbents, namely clay [6], activated charcoal (AC) [7], hydrated sodium calcium aluminosilicates (HSCAS) [8], and polymers for the binding of mycotoxins [9]; however, in some cases, a single adsorbent has been shown to be effective against one or two specific mycotoxins. The efficacy of adsorption depends on the functional groups on the surface, the physical qualities of the adsorbent, and the physical and chemical characteristics of the mycotoxins [10]. The scientific and health interest in the mentioned issues has grown during the last few years, resulting in new research on the efficiency and simultaneous use of adsorbents used for the removal of mycotoxins.

Chitosan (CTS) is a rich biopolymer attained from alkaline *N*-deacetylation of chitin, which is considered the second most copious natural biopolymer, with no harmful byproducts [11]. Its hydrophilicity, biocompatibility and high effectivity have led to a diverse range of applications in different industries [12]. CTS has shown high adsorption capacities resulting from its exceptional properties, specifically the presence of amino and carboxyl functional groups, which create very effective points for the anionic binding of polluted compounds in acidic solutions [13,14]. Previous studies [15,16,17] have discussed the applications, particularly the efficacy and specificity of the adsorption process, of CTS for mycotoxin detoxification.

Magnetic graphene oxide (MGO) nanocomposite has arisen as one of the next generation of resources with versatile applications for the removal of various toxic elements and compounds (organic and inorganic) [18]. The separation of MGO under a peripheral magnetic field can expedite the extraction of adsorbents. Magnetic separation requires less energy but achieves improved separation (particularly adsorbents with insignificant particle sizes) compared to traditional approaches such as filtration, centrifugation and gravitational separation [19]. Alteration of MGO by chemical techniques—for instance, addition of new functional groups and polymer grafting—can significantly improve the adsorption capacities of MGO. In fact, a literature study shows that no survey has been performed concerning the modification of MGO to increase the adsorption capacity of mycotoxins.

The favourable adsorption properties of MGO and the inherent properties of CTS suggest the possibility of a MGO-CTS composite as an adsorbent. The carboxyl group of MGO can chemically react with the amine group of CTS through chemical bonding between MGO and CTS [20].

The aim of this study was to investigate the effects of pH, initial mycotoxin concentrations, time, and temperature on mycotoxins adsorption. To the best of our knowledge, this is the first report of the detoxification of mycotoxins with MGO-CTS.

## 2. Results and Discussion

### 2.1. Characterisation of MGO-CTS

Numerous characterisation methods including FTIR, XRD and SEM were used to understand the behaviour and properties of MGO-CTS as a new adsorbent by examining its physical, chemical and structural properties.

**Figure 1:**
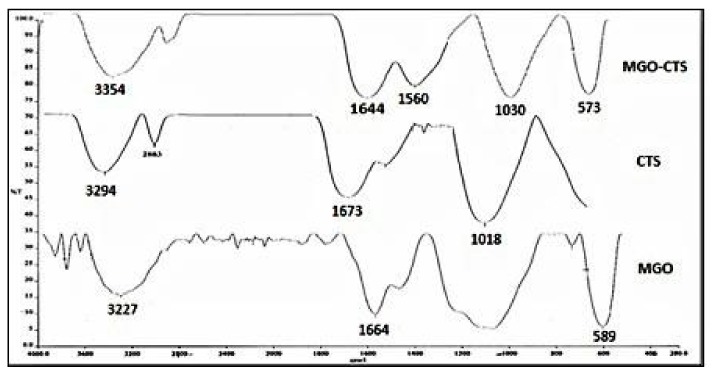
FTIR spectra of Magnetic graphene oxide (MGO), Chitosan (CTS) and MGO modified with chitosan MGO-CTS.

FTIR is one of the primary and commonly used analytical procedures to obtain information about the accessibility of certain functional groups on the surface of MGO-CTS and determine the binding mechanism of pollutants (both organic and inorganic) [21]. The FTIR spectra of all the studied compounds are shown in Figure 1. The MGO high points are located at 1664 cm^−1^ and 589 cm^−1^, related to epoxy groups, and indicate the symmetric stretching of the iron oxide (Fe_3_O_4_) nanoparticles [22]. The wide band at 3294 cm^−1^ in the FTIR spectra of CTS included the stretching vibration of the hydroxyl group and N–H bond, while the peaks at 1673 cm^−1^ and 1018 cm^−1^ are attributed to C=O of the –NH=C=O bond stretching, and the C–OH bond stretching, respectively [21]. The synergistic effect in the MGO nanocomposite by adding CTS was detected from the characteristic adsorption bands centred at 1644–1560 cm^−1^ related to the amide I (C=O stretching), amide II (Schiff’s base), and –C-O stretching of the primary alcohol group in CTS, respectively. Furthermore, a peak at 573 cm^−1^ can be observed in the FTIR spectra of MGO-CTS nanocomposite, and the existence of ferrous ferric oxide was confirmed with no noticeable change [23].

**Figure 2:**
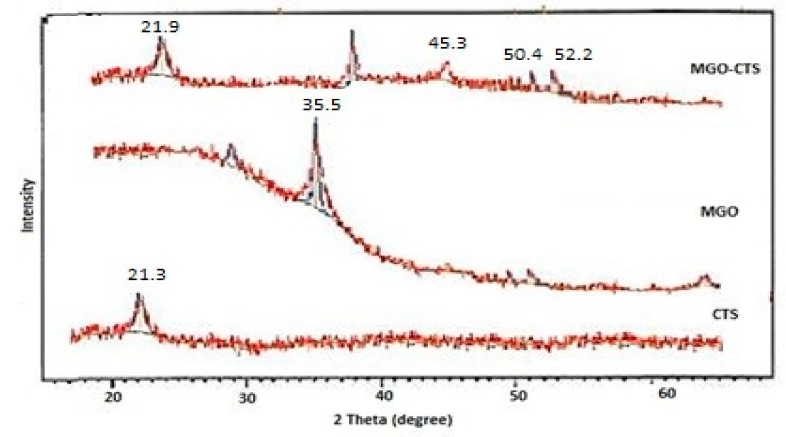
X-ray diffraction XRD patterns of the CTS, MGO and MGO-CTS.

XRD is an effective technique to determine the crystal structure of MGO-CTS by providing valuable data about the physical as well as chemical forms of MGO nanoparticles rooted in the CTS matrix. The XRD spectrograms of CTS, MGO, and MGO-CTS composites are presented in Figure 2. The pure Fe_3_O_4_ particles with spinel structures produced four distinguishing peaks for Fe_3_O_4_ (2θ = 35.5, 45.3, 50.4, and 52.2) in both samples [24]. Both the patterns of CTS and MGO-CTS (Figure 2) exhibited a broad peak at 2θ = 21.3° owing to the amorphous state of CTS, signifying that the amorphous-like structure of CTS was not altered by introduction of MGO in the nanocomposite. The results showed that magnetic composites were achieved successfully and it was not changed during the synthesis process [25].

**Figure 3:**
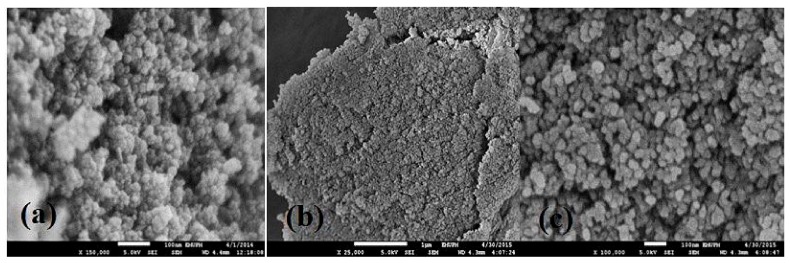
SEM images of (**a**) MGO; (**b**) CTS; and (**c**) MGO-CTS.

The morphologies and structures of CTS, MGO and MGO-CTS composites are depicted in Figure 3. As shown in Figure 3a, the SEM image of MGO illustrated the formation of magnetic nanoparticle (with regular particle size of approximately 9 nm). The typical SEM of CTS (Figure 2b) showed a tight fracture and smooth surface. The addition of MGO was successfully detected on the surface of CTS, which produced a high surface, a high density and enhanced adsorption of the active sites (Figure 3c).

### 2.2. Effect of pH on Adsorption

**Figure 4:**
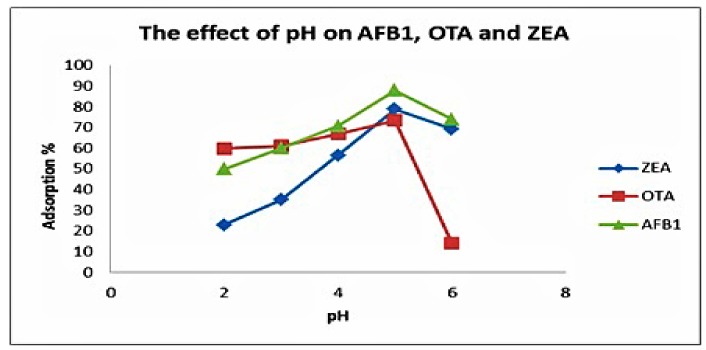
Effect of pH on the adsorption of aflatoxinB_1_ (AFB_1_), ochratoxinA (OTA) and zearalenone (ZEA) by using 0.03 g absorbent with initial concentration (50 ng/L) at 30 °C.

The effect of aqueous solution pH is a key parameter in overall adsorption procedure and impacts—the surface charge of the adsorbents as well as the speciation of the adsorbents. In the case of mycotoxins sorption behaviour, the range of pH for the batch experiments was 2–6. The adsorption increased from pH 2–5 and later displayed a sharp decrease, which might be the result of an increasing pH of the solution, as shown in Figure 4. This is normally observed because of weakened forces and, subsequently, the decrease in attraction and interaction among mycotoxin molecules and MGO-CTS [26]. In addition, the number of protonated NH_2_ groups decreased and extreme hydroxyl ions of MGO-CTS might strive with the mycotoxin anions at a pH above 5; hence, obvious reductions were observed [20]. Therefore, it can be suggested that the most optimal pH value for the decrease of most anion ions by MGO-CTS was at pH 5.0. Most of the MGO-CTS contained numerous functional groups, for instance –NH2, –NH, –COOH, –OH, Fe^+2^, and Fe^+3^, which change according to the pH of the solution. At a low pH, the majority of functional groups in MGO-CTS are protonated and positively charged. Thus, electrostatic attraction, hydrogen bonding, and π–π interactions amongst the mycotoxin molecules and the MGO-CTS surface could be the predominant adsorption mechanism.

### 2.3. Adsorption Kinetics

Adsorption is a procedure concerning liquid/solid interactions and mass transfer from the liquid surface. Adsorption kinetics can assist in predication of adsorption rates and offers valuable evidence about the mechanism of adsorption [27].

The experimental data obtained in this study were analysed by two common adsorption kinetics, namely pseudo-first-order and pseudo-second-order representations, to find the best model to describe the adsorption of the mycotoxins in PKC [28]. Towards the evaluation suitability of the diverse models, it is needed to present the correlation coefficient, *R*^2^ close or equal to 1. A higher *R*^2^ value specifies a further relevant model to the kinetics of the mycotoxins adsorption [29]. The capabilities of pseudo-first and pseudo-second order kinetic representations were examined at pH 5 in the initial concentration of the mycotoxins (50 µg/L) at a given temperature of 30 °C.

The linearised models of the pseudo-first-order and pseudo-second-order kinetic models of Lagergren are often expressed based on the following equations:

(1) $$\ln\left(q_{e}-q_{t}\right)=\ln\left(q_{e}\right)-k_{1}t$$

(2) $$\frac{t}{q_{t}}=\frac{1}{k_{2}\;q_{e}^{2}}+\frac{t}{q_{e}},$$

where *q_t_* and *q_e_* (mg/g) = the amount of solute adsorbed at time (min) and at equilibrium, respectively. *k*_1_ and *k*_2_ (min^−1^) = the pseudo-first-order rate constants for the kinetic model. The parameters are calculated from the slope and intercept of the linear plots of ln (*q_e_* − *q_t_*) vs. *t* and (*t*/*q_t_*) vs. *t*, respectively, as summarised in Table 1 [30].

According to Table 1, the correlation coefficients of the pseudo-second-order model, 0.989–0.996, are considerably higher when compared to pseudo-first-order, which was in the range of 0.661–0.945. Adsorption capacities for AFB_1_, OTA and ZEA were 3.85, 3.19 and 3.33 ng/g, respectively. Thus, it can clearly be stated that the pseudo-second-order model, suggesting a chemisorption process, can explain the interaction amongst the adsorbate and adsorbent accurately. Therefore, it can be concluded that the process obeyed chemisorption and involved valency forces through the allocation or exchange of electrons between the hydrophilic sites of adsorbent and mycotoxins. This process is irreversible because the chemically adsorbed molecules are not able to move on the surface [31].

### 2.4. Adsorption Isotherms

The adsorption isotherm is vital for describing the surface properties of the adsorbent, the capacity of an adsorption and the design of adsorption systems. Numerous other studies have been done to define the equilibrium of adsorption systems. We used two of the most common models (Langmuir and Freundlich) employed to define the adsorption behaviour of mycotoxins at different initial concentrations (5–100 ng/g).

The Langmuir isotherm is an empirical model for building a monolayer on the surface that contains a finite quantity of localised sites; all the adsorption sites on the adsorbent take place at precise homogeneous sites within the material [32].

The general form of the Langmuir isotherm is presented by the equation given below:

(3) $$q_{e}=\frac{q_{max}K_{L}C_{e}}{1+K_{L}C_{e}}$$

Based on this isotherm equation, once a template inhabits a site, no additional sorption can occur at that site. Hence, all sites are dynamically alike and no interactions amongst the molecules adsorbed on neighbouring sites occur [33].

Equation (4) can be represented in a linear form as well:

(4) $$\frac{C_{e}}{q_{e}}=\;\frac{C_{e}}{q_{max}}+\frac{1}{K_{L}q_{max}},$$

where $q_{e}$ is the number of mycotoxins per unit weight of adsorbent (ng/g), $q_{max}$ is the maximum adsorption capacity (ng/g), $C_{e}$ is the equilibrium concentration in solution, and $K_{L}$ is a constant relating to the energy of sorption (L/ng). The values of $q_{max}$ and $K_{L}$ can be evaluated from the linear plot of $C_{e}$/$q_{e}$ against $C_{e}$. This design provides a straight line with the slope and intercept equal to 1/$q_{max}$ and (1/$K_{L}q_{max}$), respectively [34].

The empirical Freundlich model applies to multilayer adsorption by non-uniform circulation of adsorption temperature and attractions over the heterogeneous surface. The Freundlich isotherm represents the proper sorption data on heterogeneous surfaces at low and intermediate concentrations [35], which is stated by the following equation:

(5) $$q_{e}=K_{F}{C_{e}}^{1/n}$$

**Figure 5:**
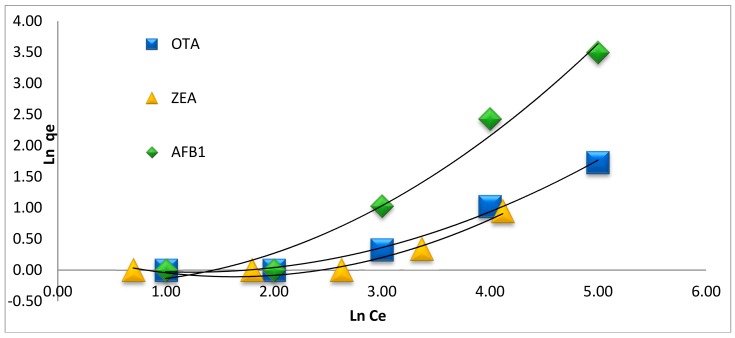
Freundlich isotherms for the adsorption of AFB1, OTA and ZEA with MGO-CTS at 50 °C.

Equation (6) can be expressed in linear form as well:

(6) $$\ln q_{e}=\;\ln K_{F}+\frac{1}{n}\;\ln C_{e},$$

where *K_F_* and *n* are the Freundlich constants, indicating the adsorption capacity and the degree of heterogeneity of the sorbent surface, respectively. A plot of ln *q_e_* versus Ln *Ce* provides a straight line with a slope of 1/*n* and an intercept equal to Ln *K_F_*, as reported in Figure 5 and Table 2 [34]. Progressively larger *K_F_* values indicate larger adsorption capacity [36]. The value of *n* is not only the amount of deviation from linearity; nevertheless, it informs us about the heterogeneity degree of the sorption sites. If *n* lies below 1 it indicates a favourable sorption process. Additionally, the isotherms with *n* < 1 are categorised as isotherms with high attraction amongst the adsorbate and the adsorbent, which is considered an indication of chemisorption and proof of an increase in hydrophobic surface properties subsequent monolayer [36,37].

The applicability of the isotherm equation is equated by finding *R*^2^ from the Langmuir and Freundlich adsorption constants. The corresponding *R*^2^ values are listed in Table 2, in which the *R*^2^ obtained using the Freundlich equation were higher than those calculated using Langmuir. On the other hand, the value of *n* < 1 indicates a favourable and stronger interaction between the mycotoxins and MGO-CTS. The values of *n* for all three mycotoxins were below 1 at 50 °C, thus indicating more interaction between the mycotoxins and MGO-CTS (Table 2).

A literature survey reported that the maximum amounts of AFB1, OTA and ZEA adsorbed by cross-linked chitosan and grape pomace were (5.67, 24.8 and 9.18 mg/g) and (15.0, 6.3 and 8.6 mg/g), respectively [37,38]. On the other hand, studies by Merdivan et al. and Trentin et al. have shown that the adsorption capacity of AFB1 and OTA was 0.73 and 2.66 mg/g, respectively [15,39],. Moreover, our team investigated the *Q_max_* of ZEA = 5.4 mg/g using MGO [40]. The current study has shown that MGO-CTS for the first time is used as an adsorbent for the reduction of AFB1, OTA and ZEA. The predicted maximum adsorption capacity (*Q_max_*) was 31.1, 75.4 and 27.7 mg/g, respectively. In comparison, MGO-CTS had higher adsorption capacity for three toxins. The results indicated that MGO-CTS would be a very promising adsorbent due to the large surface area and different functional groups.

#### Thermodynamics

Thermodynamic adsorption research offers additional data concerning the practicability and spontaneity of the adsorption procedure. The thermodynamic factors for the adsorption of mycotoxins, for instance the modification in Gibbs free energy (∆*G*°, kJ/mol), enthalpy (∆*H*°, kJ/mol) and entropy (∆*S*°, J/mol K), were estimated at various temperatures using the following equations:

(7) $$\Delta G{}^{\circ}=-RT\ln K_{c}$$

Δ*G*° = Δ*H*° − *T*Δ*S*° (8)

**Figure 6:**
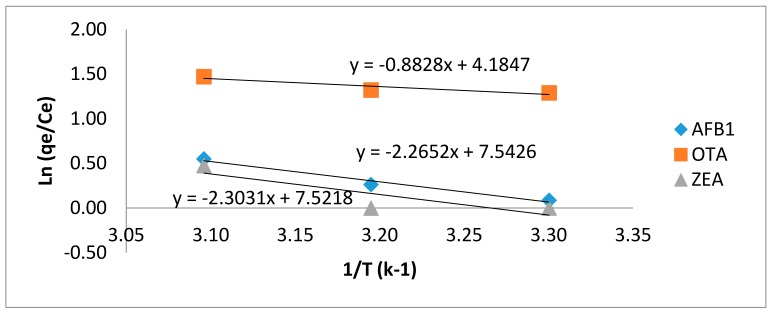
Van’t Hoff plot for assessment of thermodynamic parameters.

By combining Equations (7) and (8) we obtain:

(9) $$\ln(\frac{q_{e}}{C_{e}})=\frac{\Delta S}{R}-\frac{\Delta H}{RT},$$

where R (8.3145 J/mol K) is the universal gas constant, T is the absolute temperature (in Kelvin) and *K_c_* ($q_{e}/C_{e}$) is the adsorption affinity. Referring to Van’t Hoff in Equation (9), the values of Δ*H*° and Δ*S*° evaluated from plotting ln *K_c_* against 1/T provide a straight line with a slope and intercept equal to (−Δ*H*°/*R*) and (Δ*S*°/*R*), respectively (Figure 6). Δ*G*° was calculated from Equation (8) a procedure conferring to Equation (8) [8].

As shown in Table 3, the values of Δ*G*° for AFB_1_, OTA, and ZEA at various temperatures were negative. Henceforth, the adsorption of mycotoxins on MGO-CTS was spontaneous, while the positive Δ*S*° changes produced increased randomness during the adsorption process. From the results, the positive values of Δ*H*° show that this process was endothermic. The relatively high values of Δ*H*° may be due to the formation of strong chemical bonds between the mycotoxin molecules and functional groups of the adsorbent surface.

## 3. Conclusions

MGO-CTS was successfully produced, characterised and used as a new adsorbent for the simultaneous reduction of mycotoxins. Our results indicate that the adsorption of these mycotoxins on the surface of MGO-CTS depends on several parameters such as pH, contact time, adsorbate concentration and temperature. Analysis of adsorption mechanisms showed that the hydroxyl groups, amine and iron ions (Fe^+2^ and Fe^+3^) of CTS and MGO are predominantly responsible for binding mycotoxins. The adsorption isotherms and kinetics were calculated in detail. The equilibrium data provide the best fit using the Freundlich model by illustrating multilayer adsorption. The pseudo-second-order kinetic model successfully described the sorption reaction of the mycotoxins, demonstrating that chemisorption is the rate-controlling procedure. According to the negative value of Δ*G*°, the adsorption was spontaneous, while the positive values of Δ*S*° illustrated the amplified randomness at the solid and liquid crossing point. The adsorption of AFB_1_, OTA and ZEA onto MGO-CTS increased with increasing temperature, suggesting that the increase of temperature from 30 to 50 °C caused an increase in the adsorption capacity. Therefore, the adsorption process was endothermic. MGO-CTS as an adsorbent has promising characteristics for future applications, such as high sensitivity and simple and eco-friendly reduction of mycotoxins in environmental samples. MGO-CTS has a potentially efficient capacity for AFB_1_, OTA, and ZEA at 50 °C and pH 5. It can be concluded here that MGO-CTS can be reduced to AFB_1_, OTA, and ZEA at 50 °C and pH 5.

## 4. Materials and Methods

### 4.1. Chemicals

Palm kernel cakes (PKC) are attained from numerous local factories in diverse districts (Kelantan and Shah Alam) of Malaysia. PKC was crushed to a sufficient powder and sieved with mesh size of 750 mm before being used for spiked with mycotoxins. Chitosan (CTS > 85% deacetylation), graphite powder, sodium hydroxide (NaOH) and sodium chloride (NaCl) were provided by Sigma-Aldrich (St. Louis, MO, USA). Analytical pure standards (AFB_1_, OTA, and ZEA), AOZ immunoaffinity columns (IAC), and the phosphate-buffered saline solution (PBS) were prepared by dissolving PBS in distilled water provided by VICAM (Watertown, MA, USA). Glass microfibre filters (GF/A grade) and fluted filter papers (24 cm) were acquired from Whatman (Maidstone, UK). Ferrous ammonium sulphate [(NH_4_)_2_SO_4_FeSO_4._6H_2_O], ammonium ferric sulphate [NH_4_Fe(SO_4_)_2_∙12H_2_O], sodium nitrate (NaNO_3_), sulfuric acid (H_2_SO_4_) 98%, ethanol (C_2_H_6_O), nitric acid (HNO_3_), potassium permanganate (KMnO_4_), hydrogen peroxide (H_2_O_2_), methanol (CH_3_OH) and acetic acid (CH_3_COOH) (HPLC grade) were purchased from Merck (Darmstadt, Germany).

### 4.2. Instrumentation

The following instruments were used for data collection of the adsorbent: (i) distilled water obtained from a Milli-Q purification system (Bedford, MA, USA); (ii) adjustable pipettes (Gilson Pipetman L, Paris, France); (iii) a nitrogen evaporator with a heated block (N-EVAP, Organomation Associates, Inc., Berlin, MA, USA); (iv) a Waters high-performance liquid chromatography (HPLC) system (Milford, MA, USA); (v) an incubator shaker (New Brunswick Scientific C24, Edison, NJ, USA); (vi) Centrifuge (Sigma 3–18s Darmstadt, Germany); (vii) a vortex mixer (Harmony, Tokyo, Japan).

### 4.3. Synthesis of Adsorbents

Three adsorbents were prepared: (i) graphite oxide (GO); (ii) MGO; and (iii) MGO modified with CTS nanocomposite (MGO-CTS).

GO was prepared using a modified Hummers method [41]. GO was produced by vigorously stirring 5 g of commercial graphite powder and 2.5 g of NaNO_3_ in 75 mL of concentrated H_2_SO_4_ (98%) in an ice bath at 0 ± 2 °C. After stirring, 15 g of KMnO_4_ were gradually added, and the frequency of addition was measured to avoid an unexpected rise in the temperature. Afterwards, the mixture was preserved at 35 °C for 30 min. Next, 230 mL of distilled water were gradually added to the reaction container, keeping the temperature below 98 ± 2 °C for 15 min. Then, 700 mL of distilled water and a consequent addition of 2.5 mL of hydrogen peroxide (30 wt %) were added to the mixture. The consequential combination was filtered and cleansed three times using ultrapure water and alcohol, prior to drying at 70 °C for 12 h in a vacuum oven.

MGO was formed by a chemical co-precipitation method [42]. The iron (III) oxide (Fe_2_O_3_) nanoparticles were set by dissolving 5.8 g of (NH_4_)_2_SO_4_FeSO_4_·6H_2_O in 10.7 g of NH_4_Fe(SO_4_)_2_·12H_2_O in 100 mL of ultrapure water to produce a mixed iron salt solution under nitrogen gas. Then and there, the chemical precipitation was attained by the addition of 75 mL NH_4_OH solution (29.6%) dropwise for 30 min at 25 °C. A black precipitate instantly presented after the addition of the NH_4_OH solution. The precipitated particles are covalently bonded to carboxyl group of GO and Fe–O of iron oxide nanoparticles. Typically, 1 g of dried GO was dissolved into 100 mL of ultrapure water with ultra-sonication to produce a steady suspension. Lastly, the MGO solid was composed via a magnet, washed three times with ultrapure water and anhydrous C_2_H_6_O, and dried at 70 °C for 12 h in a vacuum oven.

The MGO-CTS composite was prepared via the following procedure. The CTS solution was arranged by dissolving 0.4 g of powder CTS (2% *w*/*v*) into 20 mL of a CH_3_COOH solution (2% *w*/*v*) and stirred for 2 h at room temperature, followed by the addition of 0.3 g of MGO to the prepared solution. The mixed solution was stirred uninterruptedly for 90 min in a water bath at 50 ± 2 °C. The pH of the solution was adjusted to 9–10 by micro-additions of NaOH (0.1 mol/L) and it was kept in a water bath for another 60 min at 80 ± 2 °C. The black product was washed with C_2_H_6_O and purified water until the pH reached approximately 7, and then it was dried in a vacuum oven at 50 °C to produce the concluding product, MGO-CTS. The arranged products (GO, MGO, and MGO-CTS) were ground to a fine powder, sized 75–125 mm, after sieving.

### 4.4. Characterisation Techniques

The XRD forms were recorded on an XRD diffractometer (model Richard Seifert 3003 TT, Ahrensburg, Germany) with a CuKα radiation for the crystalline phase identification (*k* = 0.15405 nm for CuKα). The FTIR spectrophotometer Nicolet 6700 (Thermo Nicolet Corp., Madison, WI, USA) was used to attain the FTIR spectra of MGO, CTS and MGO-CTS. The SEM (LEO 1455 VPSEM, Kensington, UK) was applied to produce an image of the material in order to find the surface morphology.

### 4.5. Adsorption Experiments

Batch adsorption of AFB_1_, OTA and ZEA was carried out using MGO-CTS as the adsorbent. For the purpose of kinetic studies, the same amount of mycotoxin was used and kept in contact with the standard solution, which was diluted in water and methanol (50:50; *v*/*v*) at each time interval (3, 4, 5, 6, 7 and 8 h) within the optimum pH and room temperature. To examine the adsorption isotherm, 5 g of PKC were spiked with AFB_1_, OTA and ZEA at three diverse concentrations (5.0, 25.0 and 100.0 ng/g). Each spike was done three times. The sampled spikes were stored in the dark overnight to permit solvent evaporation. In the preliminary study, the total amount of MGO-CTS, from 0.005 to 0.03 g, was studied; however, the decrease of mycotoxins after 0.03 g was insignificant. Henceforth, consistent with this preliminary study, the sample was agitated with 0.03 g of MGO-CTS, and an additional 20 mL distilled of methanol and water (80/20; *v*/*v*) were added. The suspensions were shaken in an incubator shaker to keep the temperature constant at 25 °C. The initial pH values were attuned with small amounts of 0.1 M HNO_3_. The agitation speed and incubation time were 160 rpm and 24 h, respectively. After the adsorption procedure, MGO-CTS was separated by magnetic separation, the supernatant was analysed for residual mycotoxin by HPLC [43] and the adsorption capacity of MGO-CTS for the mycotoxins was evaluated by the following equation:

(10) $$q_{e}=\frac{\left(C_{0}-C_{e}\right)v}{m},$$

where $C_{0}$ (µg/L) and $C_{e}$ (µg/L) are the primary and equilibrium concentratis of the mycotoxins, respectively; v (L) is the volume of the mycotoxins solution; and m (g) is the mass of adsorbent [44]. To study the thermodynamic adsorption, 5 g of PKC was spiked with mycotoxins at the highest concentration (100.0 ng/g). Thus, the effect of temperature on equilibrium was investigated at 30–50 °C.

## Figures and Tables

**Table 1:** Kinetics parameters for mycotoxins adsorption onto MGO-CTS.

Mycotoxin	Pseudo-First-Order			Pseudo-Second-Order		
	$Q_{t}=Q_{e}\left(1-e^{-k1t}\right)$			$Q_{t}=Q_{e}\left[1-\left(\frac{1}{1+K_{2}Q_{e}t}\right)\right]$		
	*k*_1_ (1/min)	*q_e,cal_* (ng/g)	*R* ^2^	*k*_2_ (g/ng min)	*q_e,cal_* (ng/g)	*R* ^2^
AFB_1_	0.002	2.45	0.928	0.002	3.85	0.989
ZEA	0.002	1.95	0.945	0.005	3.19	0.999
OTA	0.0008	1.59	0.661	0.03	3.33	0.996

AFB_1_: aflatoxinB_1_; OTA: ochratoxinA; ZEA: zearalenone.

**Table 2:** Langmuir and Freundlich isotherm factors for AFB1, OTA and ZEA on MGO-CTS (pH = 5, *t* = 6 h).

Mycotoxin	Langmuir Equation				Freundlich Equation		
	*T*	*q_max_*	*K_L_*	*R* ^2^	*K_F_*	*n*	*R* ^2^
	(°C)	(ng/g)	(L/ng)		(L/ng)		
AFB1	30	9.62	0.04	0.89	1.39	1.35	0.993
	40	7.35	0.1	0.95	2.26	1.27	0.985
	50	10.64	0.05	0.97	6.57	0.74	0.99
ZEA	30	7.25	0.02	0.99	8.08	1.25	0.994
	40	5	0.02	0.99	5.31	1.28	0.994
	50	23.26	0.53	0.945	10.93	0.66	0.988
OTA	30	72.46	0.005	0.915	3.32	1.03	0.993
	40	11.11	0.05	0.951	1.58	1.87	0.995
	50	27.02	0.01	0.912	4.58	0.94	0.993

**Table 3:** Thermodynamic factors for the adsorption of mycotoxins onto MGO-CTS.

Mycotoxin	*C*_0_	T (°C)	*q_e_*	*K_c_*	Δ*G*°	Δ*H*°	Δ*S*°
AFB1	100	30	6.28	0.09	−18.97	18.78	62.68
	100	40	6.34	0.26	−19.60		
	100	50	6.42	0.55	−20.22		
OTA	100	30	56.63	1.29	−10.52	7.31	34.75
	100	40	62.22	1.32	−10.87		
	100	50	81.35	1.47	−11.22		
ZEA	100	30	2.69	−3.10	−49.52	51.62	163.61
	100	40	3.67	−2.51	−57.43		
	100	50	4.71	−1.83	−59.06		
